# Terahertz Spectroscopy in Assessing Temperature-Shock Effects on Citrus

**DOI:** 10.3390/s24227315

**Published:** 2024-11-15

**Authors:** Junbo Wang, Ziyi Zang, Xiaomei Li, Dongyun Tang, Qi Xiao, Mingkun Zhang, Shihan Yan

**Affiliations:** 1 Chongqing Institute of Green and Intelligent Technology, Chinese Academy of Sciences, Chongqing 400714, China; wangjunbo@cigit.ac.cn (J.W.); lixiaomei@cigit.ac.cn (X.L.); tangdongyun@cigit.ac.cn (D.T.); 2 Chongqing School, University of Chinese Academy of Sciences, Chongqing 400714, China; 3 Aerospace Times FeiHong Technology Company Limited, Beijing 100094, China; zangziyi_l@163.com; 4 Wanzhou Institute for Food and Drug Control, Chongqing Key Laboratory of Development and Utilization of Genuine Medicinal Materials in Three Gorges Reservoir Area, Chongqing 404000, China; xq1600818606@163.com

**Keywords:** THz spectroscopy, sensor, plant, high temperature, water deficit

## Abstract

Rapid assessment of physiological status is a precondition for addressing biological stress in trees so that they may recover. Environmental stress can cause water deficit in plants, while terahertz (THz) spectroscopy is sensitive to changes in aqueous solutions within organisms. This has given the THz sensor a competitive edge for evaluating plant phenotypes, especially under similar environmental stress, if there are existing differences in the corresponding THz information. In this study, we utilized THz technology in association with traditional weighing methods to explore physiological changes in citrus leaves under different temperature, duration, and stress treatment conditions. It was found that the higher the temperature and the longer the exposure duration, the more severe the reduction in the relative absorption coefficient. There was a positive correlation between the trends and the increase in the ion permeability of cells. In addition, based on the effective medium theory, THz spectral information can be transformed into information on free water and bound water in the leaves. Under different treatment conditions, water content shows different trends and degrees of change on the time scale, and accuracy was verified by traditional weighing methods. These findings revealed that characteristics of THz information can serve as a simple and clear indicator for judging a plant’s physiological status.

## 1. Introduction

The increasing regularity of extreme weather events has exposed risks to agricultural production, especially for economic crops such as fruits. The repair cycle for woody perennials after experiencing external stresses is typically several years long [1]. Early diagnostic methods can help intervene promptly, thereby optimizing production management, promoting rehabilitation, and reducing economic losses [2,3,4]. In this process, the sensitivity and accuracy of sensors are equally important. Environmental stress usually causes a water deficit and has a great impact on plant yield. In recent years, considering the crucial role of water in life [5,6] and leaves as direct environmental sensors in plants, monitoring their water status has been proven to be a direct and effective way to assess their physiological status [7,8,9].

The development of spectral detection technology, which features contactless, label-free, and real-time detection, has accelerated progress in identifying disaster situations [10,11,12]. An example is a terahertz (THz)-based detection technique that has attracted considerable attention due to its natural water sensitivity [13,14]. Water has a strong THz absorption coefficient, the numerical value of which is typically an order of magnitude higher than other biomolecules such as proteins, nucleic acids and sugars [15,16]. It is also present to a great extent in living organisms. Water has a strong linear relationship with changes in THz amplitude over a wide volume range [17]. Naturally, THz spectroscopy for qualitative and quantitative water measurements is increasingly used in agriculture, food science, and other fields [18]. There appears to be a strong correlation between the increased THz amplitude intensity and decreased moisture content in biological samples [19,20], and this has been supported by many plant model validation experiments [21,22,23]. There are two natural states of water in plant leaf tissues, free water and bound water. The former consists in free-moving water molecules that provide transport and a medium for biochemical reactions, while the latter consists in hydrated water molecules that exist in the space of macromolecular structures and are hardly involved in metabolism [24]. Moreover, the differences in hydrogen bonds and other low-frequency vibrations between free water and bound water molecules in the THz band enable depiction of the changes in plant water status under stress conditions in detail [25,26,27]. Recently, we analyzed the THz spectral characteristics corresponding to the physiological and biochemical properties of leaves under cold stress and found different responses of leaves to chilling or freezing [28]. However, we cannot ignore the fact that plants generally use similar measures to cope with the challenge of water scarcity when facing diverse stress conditions. For example, under adverse conditions, an increase in the ratio of bound water content to free water content is a common coping strategy among plants [29,30]. Considering the close correlation between THz information and water state changes [21,31], similar physiological development pathways pose challenges for early monitoring based on the THz method.

In this study, we used citrus as the detection object, because it is one of the foremost fruits globally and is also susceptible to abrupt elevations in temperature. A high-temperature stress model was constructed using 30 °C, 35 °C, and 40 °C treatments and analyzed by THz spectroscopy in comparison with normal-growth-state plants to evaluate the sensitivity and accuracy of THz schemes. The results demonstrated distinct trends in relative THz absorption coefficients appearing under different-temperature stress treatments, and the strength of the changes corresponded to the degree of stress. Through quantitative analysis of free and bound water changes based on the effective medium theory, it was observed that the volume fraction of free water decreased at 30 °C, 35 °C, and 40 °C, while the volume fraction of bound water exhibited a corresponding pattern of remaining constant at 30 °C and increasing at 35 °C and 40 °C. Moreover, changes in damage rate and water status of leaf cell membranes showed a direct correlation with THz spectra, assessed through ion permeability detection and gravimetric methods. By comparison with previous THz spectral studies on citrus leaves conducted under different low-temperature treatment conditions, the THz spectral information of plants under water stress corresponding to the characteristics of different physiological states varies. Based on this, we reveal the feasibility of employing THz time-domain spectroscopy (THz-TDS) to monitor high-temperature stress in plants at various levels, suggesting its potential for early diagnosis.

## 2. Materials and Methods

### 2.1. Plant Materials and Treatment

The experimental material came from a monoclonal population of Citrus sinensis (Linn.) Osbeck cv. Huihong Long, which is cultivated in the experimental field of the Citrus Research Institute of Southwest University, situated in Chongqing, China. The model sample contained 20 individuals of similar growth, avoiding experimental errors caused by genetic and phenotypic differences as much as possible.

Plant materials in their natural growth state were used as the control group. In the treatment group, plant materials underwent high-temperature treatment within an incubator (GZX-9023MBE, Boxun, Shanghai, China) at predetermined time and temperature parameters. The treatment conditions comprised: (1) consecutive exposure durations of 1, 2, 4, 6, and 16 h at temperatures of 30 °C, 35 °C, and 40 °C, and (2) 1 h of high-temperature exposure at 30 °C, 35 °C, and 40 °C, followed by 1 h rest at room temperature, for 5 cycles. In addition, another batch of blades treated under the same conditions was allocated for electrolyte leakage rate testing (DDSJ-308A, Leici, Shanghai, China).

Blade materials were first subjected to THz spectroscopy testing in transmission mode, with the light spot avoiding the leaf vein to reduce experimental errors caused by non-uniform structure. Subsequently, the thickness and weight of the blades were measured using a micrometer and an electronic balance (ME204E, Mettler Toledo, Shanghai, China). Multiple leaf samples were used in each experiment to obtain mean results. These samples comprised random selections at least 20 leaves from five branches of different plants with the aim of reducing potential experimental bias.

**Figure 1:**
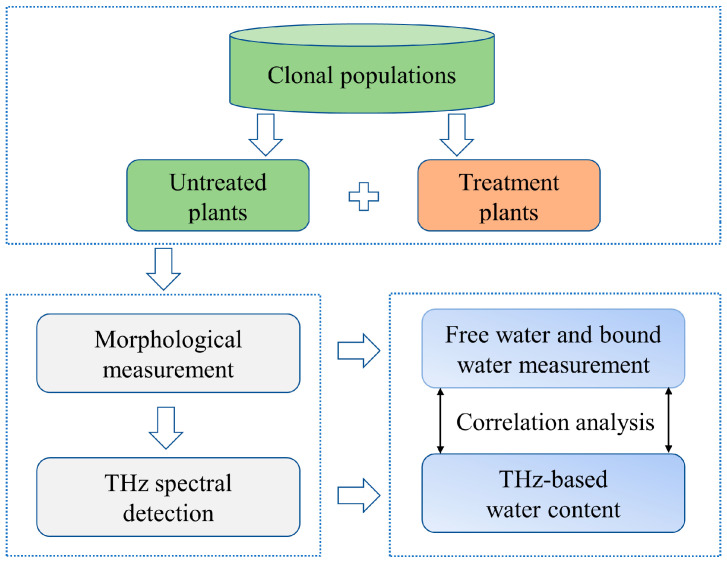
Schematic diagram of the experimental process.

The specific experimental steps are shown in Figure 1. The cloned citrus plants were planted in a constant-temperature laboratory and hydrated at the same time intervals to maintain normal vigor. Subsequently, the citrus leaves without high-temperature stress treatment and those after high-temperature treatment were subjected to morphological tests, such as weight and thickness and THz spectroscopy. The traditional weighing method combined with an Abbe refractometer was used to measure the moisture content of citrus leaves and changes therein, including the total moisture as well as free and bound water, and correlation analyses were performed to verify the scientific validity and accuracy of the THz spectroscopic information.

### 2.2. THz-TDS System

**Figure 2:**
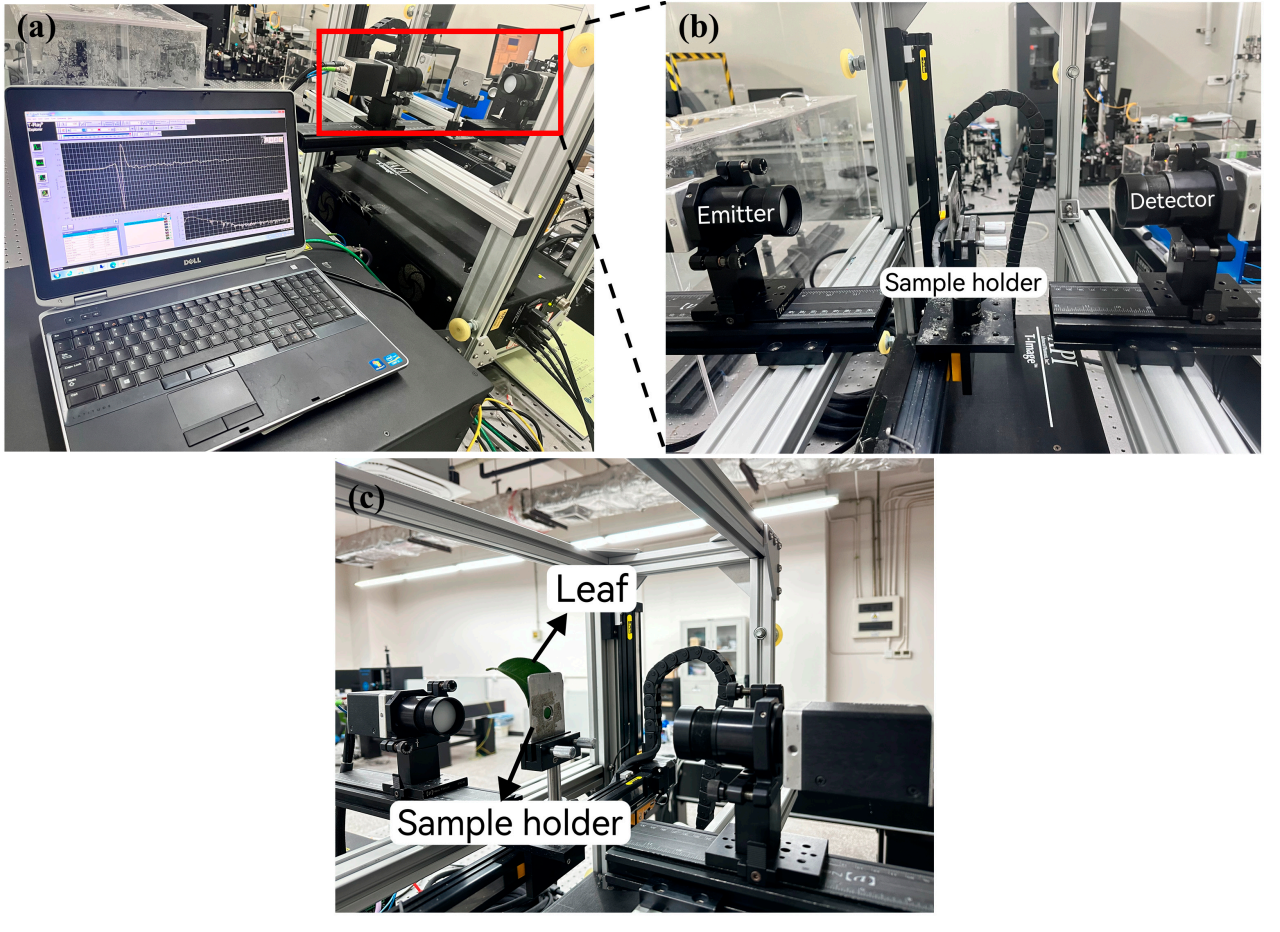
THz-TDS system (**a**), THz spectral measurement in working mode (**b**), and sample holder with leaf sample (**c**).

The THz-TDS system (T-Gauge 5000, Advanced Photonix, Inc., Camarillo, CA, USA) has a detection frequency range of 0.1 to 3.5 THz, a spectral resolution of 12.5 GHz, a scanning range of 80 ps@0.1 s, and a signal-to-noise ratio of greater than 70 dB. The THz time-domain spectroscopy system spectral measurement working mode consists of an emitter, detector, and sample holder. During the experiment, the sample holder was placed behind a translation stage and adjusted so that the sample to be measured was placed at the focal point of the THz optical path (Figure 2a,b). The THz technique has obvious advantages over traditional weighing methods in terms of detection efficiency [28].

### 2.3. THz Optical Parameter Extraction and Analytical Calculation of Data

The THz-TDS system operating in transmission mode was used to perform the THz spectrum acquisition [32]. In the collection of THz spectrum data, ten to twenty testing points in mesophyll were randomly selected. The number of spatial points was dependent on the leaf area. The absorption coefficient of leaves can be calculated by the formula [33]:

(1) $$\alpha \left(\omega \right)=-\frac{2}{d}ln\left[A\left(\omega \right)\frac{{\left(\frac{\Delta \left(\omega \right)c}{\omega d}+2\right)}^{2}}{4\left(\frac{\Delta \left(\omega \right)c}{\omega d}+1\right)}\right]$$

where symbols *A(ω)* and Δ*(ω)* represent the amplitude ratio and the phase difference, respectively, of the Fourier transforms pertaining to the electric field transmission of the leaf and the reference. Here, *ω* denotes the angular frequency, *c* stands for the speed of light, and *d* signifies the average thickness of the leaf. The thickness *d* was determined through the measurement of a minimum of fifteen points randomly distributed across the mesophyll [34].

The absorption coefficient is an important parameter that describes the degree of absorption of electromagnetic waves (e.g., THz waves) by a material. Dissipation occurs after the THz pulse is incident on the sample, and the energy transfer process from the THz wave to the sample attenuates the amplitude of the THz field and shifts its phase. Considering the strong correlation between water contents and the absorption coefficient of the testing object, we used this indicator to evaluate water content in leaves.

The absorption coefficients of leaves can be derived from Equation (1), but some differences in the initial absorption coefficients may occur due to individual differences in leaves, resulting in a lack of clarity and readability of the results. Therefore, we defined relative absorption coefficients to highlight the THz response of leaves under high-temperature stress.

(2) $$\alpha_{relative}=\frac{\alpha_{after}-\alpha_{before}}{\alpha_{before}}$$

Subsequently, we utilized a scheme based on the effective medium theory to quantitatively calculate the volume fractions of free water and bound water in the blades. This method utilizes a linear absorption model to determine the effective absorption coefficients of leaf solids and free and bound water:

(3) $$\alpha_{Leaf}=c_{FW}\alpha_{FW}+c_{BW}\alpha_{BW}+c_{S}\alpha_{S}$$

where $\alpha_{i}$ denotes the absorption coefficient, while $c_{i}$ represents the volumetric fraction of a specific component, with indices corresponding to free water (*FW*), bound water (*BW*), and solid matter (*S*) ($\sum c_{i}=1$). Here, $\alpha_{Leaf}$ was obtained by experimentation, while the $\alpha_{FW}$, $\alpha_{BW}$, and $\alpha_{S}$ parameters were obtained from our previous research [28,31]. Thus, the $c_{i}$ clues that can render the calculation results on both sides of the equation consistent can be regarded as the accurate volume fractions (*VF*) of free water, bound water, and solid within the leaf.

We also defined the relative change in volume fraction measured by THz spectroscopy based on our previous research [28]. The use of a unified data processing method to obtain the THz index has practical significance in making the comparison of results between different species and treatments more objective.

The relative change in volume fraction of a leaf is defined as:

(4) $$RVF=\frac{VF_{after}\left(\%\right)-VF_{before}\left(\%\right)\;}{VF_{before}\left(\%\right)}$$

where *VF* represents the volume fraction of free water or bound water or the ratio of the volume fraction of bound water to that of free water, all of which were calculated as the average of multiple spectral points on the leaf. The relative change in leaf volume fraction under repeated high-temperature stress is the relative change in leaf volume fraction after several rounds of repeated high-temperature stress, with the initial leaf not experiencing high-temperature stress used as a reference. The subscript denotes whether the physical quantity was measured before or after the high-temperature stress.

Changes in bound water content and the ratio of bound water content to free water content are of key interest to us when plants face stress. Therefore, under repeated high-temperature stress, we also explored the changing patterns and reasons [30].

### 2.4. Physiological Indicator Tests

To verify the scientificity and accuracy of the THz information, we used traditional weighing methods combined with an Abbe refractometer to measure the water content and changes, including total water as well as free water and bound water. The calculation formula and operation method were taken from previous reports [35,36,37].

Furthermore, we also measured the electrolyte leakage rate as an indicator the degree of cell damage. Deionized water (10 mL) was added to a test tube containing a leaf blade (6 mm in diameter and about 0.2 g in total weight) and allowed to stand in the dark at room temperature for 12 h before measuring its conductivity using a conductivity meter ($C_{1}$). Subsequently, the tubes were immersed in boiling water for 20 min and the conductivity ($C_{2}$) was recorded after cooling to room temperature. Electrolyte leakage was quantified as follows: [28]

(5) $$EL=\frac{C_{1}}{C_{2}}\times 100\%$$

### 2.5. Correlational Analysis

The correlation between THz response and physiological performance was assessed by analyzing the THz data in comparison with the electrolyte leakage rate or gravimetric method results and calculating the coefficient of determination (R^2^). The root mean square error (RMSE) was calculated as follows:

(6) $$\mathrm{RMSE}=\sqrt{\frac{\sum_{i=1}^{n}{\left(y_{i}-y_{i}^{,}\right)}^{2}}{N}}$$

where *y* is the content value estimated by the THz-based method, $y_{i}^{,}$ is the content value measured by the traditional method, *i* is the index, and *N* is the total number of leaf samples.

## 3. Results

### 3.1. Continuous High-Temperature Treatment

#### 3.1.1. THz Spectroscopy

**Figure 3:**
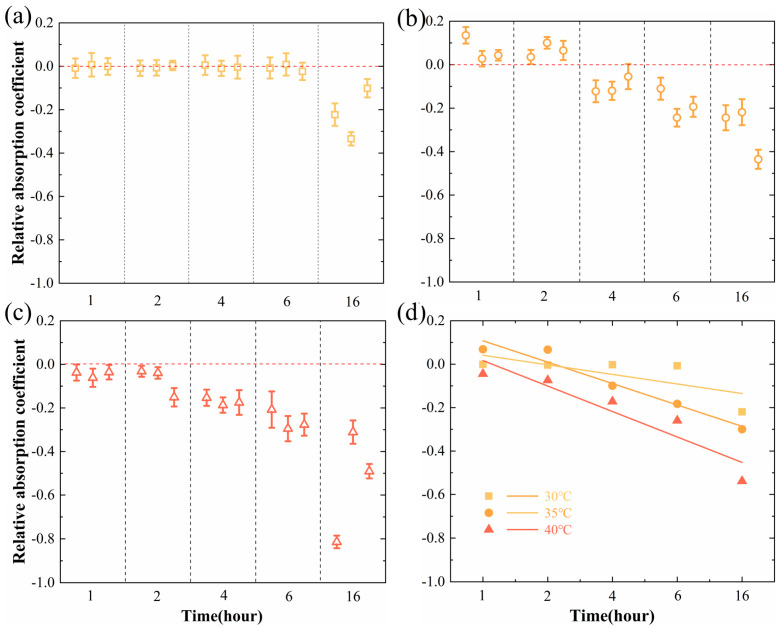
Relative absorption coefficient of leaves after high−temperature stress at 30 °C (**a**), 35 °C (**b**), 40 °C (**c**) for 1, 2, 4, 6 and 16 h, and (**d**) linear fitting curves. The original reference zero is shown as red dashed line.

The degree of heat-induced damage in citrus plants is contingent upon the intensity and duration of high-temperature exposure. According to reported results on temperature thresholds, we exposed leaves to 30 °C, 35 °C, and 40 °C for 1, 2, 4, 6, and 16 h for studying. The THz absorption coefficient of the blade varies monotonically with frequency (Figure S1), and thus the average value of a certain frequency parameter can be used to represent the overall trend of change. Based on Formula (2), Figure 3 shows the variation in the relative absorption coefficient at 0.7 THz over time after exposure to temperatures of 30 °C, 35 °C, and 40 °C for 1, 2, 4, 6, and 16 h. Under the condition of 30 °C treatment, the relative absorption coefficient of citrus leaves remained relatively unchanged for the first 6 h, until a 20% change was observed after 16 h of exposure (Figure 3a). By comparison, under the treatment condition of 35 °C, the decrease in THz relative absorption coefficient occurred earlier and the change was more significant (Figure 3b). The treatment at 40 °C directly led to a decrease in the relative absorption coefficient within 1 h, and the trend of change over time was sharper than that of the treatment at 35 °C (Figure 3c). Overall, high-temperature treatment reduced the THz absorption coefficient of citrus leaves and that trend became more pronounced with increasing temperature and time (Figure 3d).

**Figure 4:**
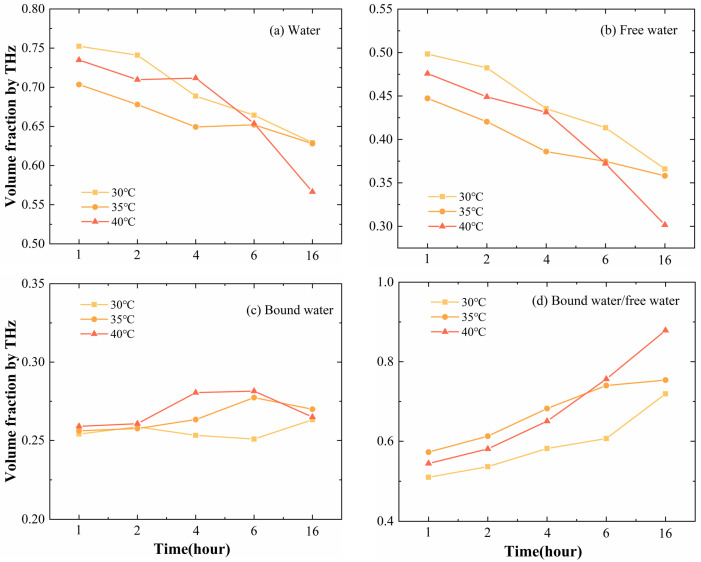
Volume fraction of water, free water, and bound water and the ratio of bound water and free water in leaves after high-temperature stress at 30 °C, 35 °C, and 40 °C for 1, 2, 4, 6, and 16 h based on THz spectroscopy.

Quantitative analysis helps eliminate background factors and individual differences in research subjects and helps decision-makers make scientific and effective judgments. The change in the ratio between free and bound water is an important indicator of the physiological state of a plant. THz spectroscopy combined with effective medium theory showed us the quantitative water state changes in plants under high-temperature stress. Overall, different degrees of high-temperature treatment all led to a decrease in the water content of plant leaves (Figure 4a). The performance of free water was consistent with the trend of overall water changes and decreased (Figure 4b). On the other hand, the changes in bound water content exhibited more diversity at different temperature states. Before and after treatment at 30 °C, the bound water content of citrus leaves remained the same. Under higher-temperature heat stress, there was an increasing trend (Figure 4c). Although the trends of change in the content of free water and bound water were different, they all led to a rise in the ratio of bound water to free water (Figure 4d).

#### 3.1.2. Ion Leakage Rate

**Figure 5:**
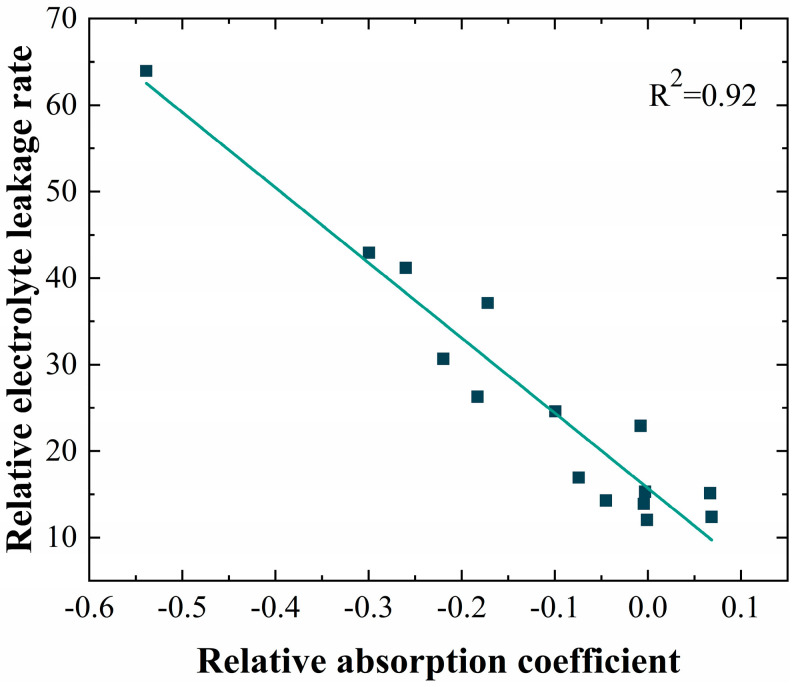
Correlation analysis of relative electrolyte leakage rate and relative absorption coefficient.

To further demonstrate the relationship between THz response and stress state, we investigated the changes in ion leakage rate. When citrus leaves were subjected to high-temperature stress, the increase in or even destruction of cell membrane permeability led to a large amount of electrolyte leakage from the cells, which increased the relative conductivity of the cellular extracts, and this phenomenon became more and more evident with the increase in the degree and duration of high-temperature stress (Table 1). Through correlation analysis, it was found that the decrease in relative absorption coefficients of THz spectra under different high-temperature conditions had a high correlation with the increase in relative ion leakage rate (Figure 5). This relationship can be regarded as direct evidence of the THz response of leaves under abiotic stress being related to cell damage.

#### 3.1.3. Gravimetric Method

**Figure 6:**
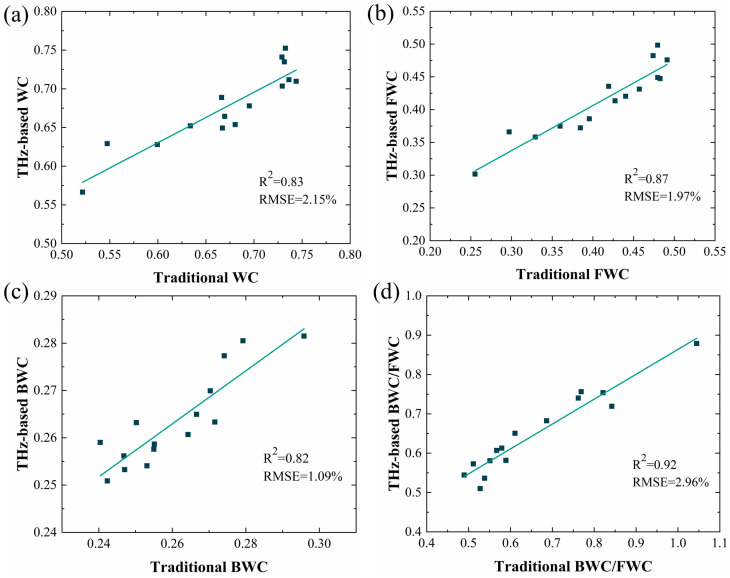
Correlation analysis of leaf WC (**a**), FWC (**b**), BWC (**c**), and BWC/FWC (**d**) content measured using THz spectroscopy and gravimetric methods.

We once again validated the analytical capability of this scheme through traditional gold indicators. We determined the content of free and bound water using the gravimetric method in combination with an Abbe refractometer. In addition to the identified trends in total water content, we plotted linear fitting curves of the changes in mass fraction and volume fraction of conventional water under different procedures (Figures S2 and S3). The determining values under the conventional methods were compared to THz information, and a strong linear positive correlation was found between results obtained by these two methods for water content (Figure 6a), free water content (Figure 6b), bound water content (Figure 6c), and the ratio of bound water/free water (Figure 6d).

Furthermore, a comparison of water state changes in citrus under different low- or high-temperature stress analyzed by THz spectroscopy and conventional methods is presented in Table 2 [28]. Although plants were all in a water-deficient state, their characteristics differed in detail. It is worth noting that under high-temperature stress, the change trend of bound water content was slightly different. Under 30 °C stress, the bound water content basically unchanged, but under 35 °C and 40 °C stress, the bound water content increased.

### 3.2. Intermittent High-Temperature Treatment

#### THz Spectroscopy

**Figure 7:**
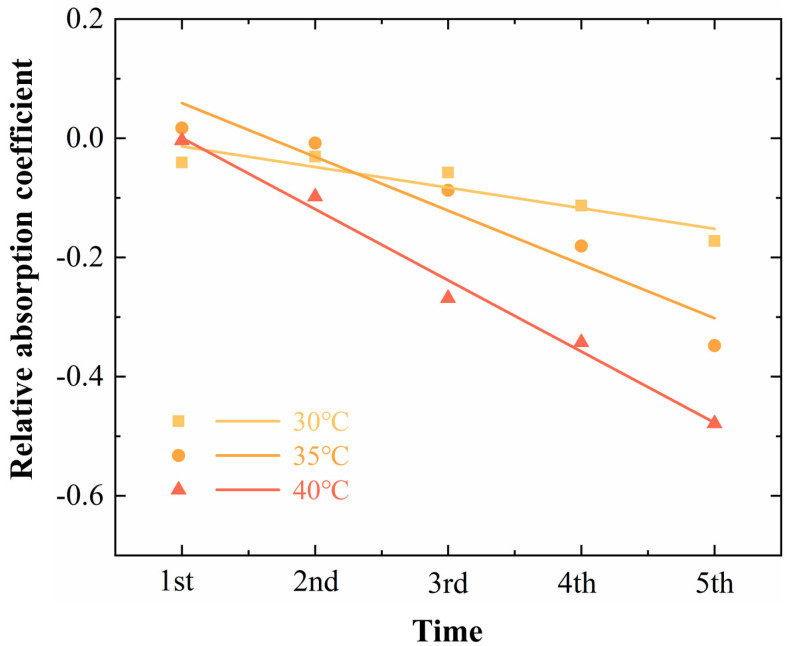
Linear fitting curves of relative absorption coefficients of leaves under five consecutive intermittent high−temperature cycles.

**Figure 8:**
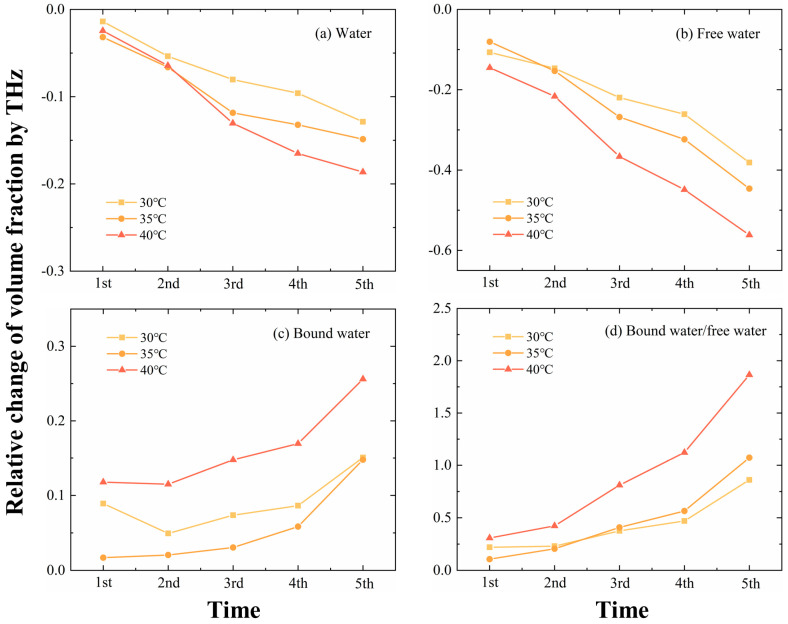
Relative change in volume fraction of water, free water, bound water, and the ratio of bound water and free water in leaves under 5 consecutive intermittent high−temperature cycles at 30 °C, 35 °C, and 40 °C using THz spectroscopy.

The occurrence and development of high-temperature stress are diverse and trigger different plant responses [38]. We conducted cyclic treatments for heating and recovery to simulate repeated high-temperature stress. In Figure 7, the results under three different conditions of 30 °C, 35 °C, and 40 °C were obtained by linear fitting. By and large, under repeated high-temperature stress, the relative absorption coefficients of leaves were smaller with the increase in the number of cycles, and it was found that the relative absorption coefficients of leaves under repeated high-temperature stress decreased more rapidly than those under continuous high-temperature stress. An interesting observation is that after the first cycle of repeated high-temperature stress at 30 °C, the relative absorption coefficient of leaves increased slightly and then also decreased gradually. Further analysis of leaf water status revealed that water and free water volume fractions decreased under all three conditions (Figure 8a,b). The changes in bound water volume fraction were slightly different, with the bound water volume fraction decreasing and then increasing with 30 °C treatment, but keeping an increasing trend with 35 °C and 40 °C treatment (Figure 8c). Although the bound water trend was not the same, the bound/free water ratios were all consistently increased (Figure 8d).

## 4. Discussion

This kind of citrus is recognized for its notable drought tolerance [39,40], but over time, long-term mild heat stress can also lead to changes in the physiological state of leaves [41,42]. The effect of high-temperature stress is usually manifested as water deficit [43]. Under environmental stress, the internal water content of crop leaves, one of the most important physiological organs of the crop itself, changes, and this is reflected in the crop’s water information status, which can be apprehended through the crop’s water status and growth status [44,45]. To ensure normal physiological activity, plants usually lower their body temperature through transpiration [46] and the proportion of bound water usually increases. By combining water with biomolecules such as proteins and cellulose to form a stable structure, the stability of the intracellular environment is increased and the impact of the external environment on biomolecules is reduced, thereby enhancing the stress resistance of organisms [47,48]. But when the pressure intensifies, transpiration instead plays a negative role, resulting in intensified dehydration and also leaf shapes changing (Figures S4–S6, Table S1) [49]. Typically, the higher the intensity of temperature shocks, the faster and more intense the reactions [50].Considering the high proportion of water in living organisms, the strong THz uptake of water, and the difference in THz dielectric constant between free water and bound water [51], the process of physical dehydration of plant leaves induced by high temperature is the direct and major cause of the THz signal changes. In addition, with the loss of water in the plant body, the permeability of the cell membrane increases, and a large amount of intracellular electrolyte leakage leads to an increase in conductivity [52,53]. An increase in ion content in aqueous solutions can also lead to a decrease in THz amplitude [54]. Changes in water status in biological samples can be sensitively measured through THz detection [55]. Due to the unique advantages of THz spectroscopy and imaging for water detection, quantitative dynamic monitoring of water content in plant leaves under different degrees of abiotic stress was realized. Combining THz indicators with cellular physiological phenomena, the above THz spectral characteristics prove that it serves as a simple and clear indicator to indicate the ability of plants subjected to different degrees of high-temperature stress.

The repeated heat stress that occurs in midsummer and early autumn is an important cause of plant resistance development, but at the same time, heat waves can also lead to plant death [56,57]. For resistant crops, an appropriate high temperature is beneficial for plant growth. With an acceleration in metabolism, the demand for intracellular and extracellular gas exchange increases, which leads to an increase in stomatal opening and gaps in mesophyll tissue, indirectly leading to a decrease in relative water content in leaves. This may be the reason for the slight increase in the relative absorption coefficient of leaves in the early stage of 30 °C treatment. At the same time, free water and bound water can be converted into each other as metabolism progresses. The acceleration of metabolism requires more free water, which will lead to a decrease in bound water content. However as the conditions become more severe, the ratio of bound water to free water increases, which is beneficial for improving the plant’s stress resistance [29]. In addition, the performance of plants under environmental stress is complex and diverse. Different plants and varieties have varying sensitivities to water stress and face varying impacts [58,59]. Intracellular heat-shock proteins and water-channel proteins are strongly enriched under repetitive high-temperature stress, resulting in more severe damage to the plant brought about by repetitive high-temperature stress than by continuous high-temperature stress [60,61], which could be the reason for the faster decrease in THz relative absorption coefficients under repetitive high temperatures. Here, THz spectral information reflects the differential physiological changes in plant leaves under various external environmental stresses [62], which is beneficial for the early diagnosis of stress states.

In summary, there were correlations between the THz response within the leaves under different temperature stresses and their corresponding physiological parameters. Moreover, THz technology has obvious advantages over the traditional weighing method in terms of simplicity, speed, and accuracy. This unique THz spectral characterization of the stress response is expected to be the basis for differentiating between different degrees of abiotic stress.

## 5. Conclusions

In this study, we determined that changes in relative THz absorption are closely related to changes in leaf water status. Higher-temperature treatment led to more water loss, and the decreasing trend of THz amplitude became more apparent. Based on the effective medium theory analysis, THz information revealed the variation characteristics in free water and bound water content of plant leaves under high-temperature stress. Of particular note, the development of this change is related to the strength of stimulation. Moreover, THz information can further identify the impact of stress cycles on plant leaves, reflecting the difference between repeated and continuous high-temperature stresses. By comparing the results of THz spectroscopy detection with physiological indicators, the results demonstrate that THz technology has distinguished the various stress states of plants, and different responses correspond to different types and degrees of stress. Therefore, Thz-based monitoring of free and bound water content has the advantage of being rapid and accurate in assessing physiological responses in plant leaves. With subsequent in-depth exploration of the biological interpretation of THz information and validation of its application in a wider range of species and scenarios, this simple and efficient method opens the door for the application of THz spectroscopy in agriculture for monitoring the response and adaptation of plants to changes in environmental factors, especially for the purposes of crop improvement and plant cultivation.

## Figures and Tables

**Table 1:** Relative electrolyte leakage rate of leaves treated at 30 °C, 35 °C, and 40 °C.

Treatment Time (h)	Relative Electrolyte Leakage Rate		
	30 °C (%)	35 °C (%)	40 °C (%)
Control (20 °C)	9.44 ± 1.27	9.44 ± 1.27	9.44 ± 1.27
1	12.03 ± 0.96	12.37 ± 0.54	14.29 ± 2.31
2	13.88 ± 3.93	15.12 ± 1.81	16.92 ± 2.51
4	15.28 ± 5.69	24.57 ± 7.94	37.11 ± 7.67
6	22.91 ± 6.01	26.27 ± 4.82	41.19 ± 8.03
16	30.66 ± 7.35	42.92 ± 8.56	63.92 ± 9.20

**Table 2:** Change trend of volume fraction of water, free water, bound water, and bound water/free water in leaves under various temperature-shock conditions.

	Volume Fraction (%)			
	Water	Free Water	Bound Water	Bound Water/Free Water
30 (°C)	↓	↓	→	↑
35 (°C)	↓	↓	↑	↑
40 (°C)	↓	↓	↑	↑
Chilling stress	↓	↓	↑	↑
Freezing stress	↑	↑	↑	↑

## Data Availability

All study data are included in the article and/or Supplementary Materials, and all raw data are available from the corresponding author upon reasonable request.

## Supplementary Materials

The following supporting information can be downloaded at https://www.mdpi.com/article/10.3390/s24227315/s1.

## References

[1] Li S., Lu S., Wang J., Chen Z., Zhang Y., Duan J., Liu P., Wang X., Guo J. (2023). Responses of Physiological, Morphological and Anatomical Traits to Abiotic Stress in Woody Plants. Forests.

[2] Ren A., Zahid A., Fan D., Yang X., Imran M.A., Alomainy A., Abbasi Q.H. (2019). State-of-the-Art in Terahertz Sensing for Food and Water Security—A Comprehensive Review. Trends Food Sci. Technol..

[3] Huang C.H., Singh G.P., Park S.H., Chua N.-H., Ram R.J., Park B.S. (2020). Early Diagnosis and Management of Nitrogen Deficiency in Plants Utilizing Raman Spectroscopy. Front. Plant Sci..

[4] Walsh J.J., Mangina E., Negrão S. (2024). Advancements in Imaging Sensors and AI for Plant Stress Detection: A Systematic Literature Review. Plant Phenomics.

[5] Sun Y., Wang C., Chen H.Y.H., Ruan H. (2020). Response of Plants to Water Stress: A Meta-Analysis. Front. Plant Sci..

[6] Lin P.-A., Kansman J., Chuang W.-P., Robert C., Erb M., Felton G.W. (2023). Water Availability and Plant-Herbivore Interactions. J. Exp. Bot..

[7] Li Z., Liu Y., Hossain O., Paul R., Yao S., Wu S., Ristaino J.B., Zhu Y., Wei Q. (2021). Real-Time Monitoring of Plant Stresses via Chemiresistive Profiling of Leaf Volatiles by a Wearable Sensor. Matter.

[8] Cecilia B., Francesca A., Dalila P., Carlo S., Antonella G., Francesco F., Marco R., Mauro C. (2022). On-Line Monitoring of Plant Water Status: Validation of a Novel Sensor Based on Photon Attenuation of Radiation through the Leaf. Sci. Total Environ..

[9] Serrano-Finetti E., Castillo E., Alejos S., León Hilario L.M. (2023). Toward Noninvasive Monitoring of Plant Leaf Water Content by Electrical Impedance Spectroscopy. Comput. Electron. Agric..

[10] Xia J., Zhang W., Zhang W., Yang Y., Hu G., Ge D., Liu H., Cao H. (2021). A Cloud Computing-Based Approach Using the Visible near-Infrared Spectrum to Classify Greenhouse Tomato Plants under Water Stress. Comput. Electron. Agric..

[11] Lassalle G. (2021). Monitoring Natural and Anthropogenic Plant Stressors by Hyperspectral Remote Sensing: Recommendations and Guidelines Based on a Meta-Review. Sci. Total Environ..

[12] Sanaeifar A., Yang C., de la Guardia M., Zhang W., Li X., He Y. (2023). Proximal Hyperspectral Sensing of Abiotic Stresses in Plants. Sci. Total Environ..

[13] Zahid A., Abbas H.T., Ren A., Zoha A., Heidari H., Shah S.A., Imran M.A., Alomainy A., Abbasi Q.H. (2019). Machine learning driven non-invasive approach of water content estimation in living plant leaves using terahertz waves. Plant Methods.

[14] Zhang Y., Wang X., Wang Y., Hu L., Wang P. (2023). Detection of Tomato Water Stress Based on Terahertz Spectroscopy. Front. Plant Sci..

[15] Wei L., Yu L., Jiaoqi H., Guorong H., Yang Z., Weiling F. (2018). Application of Terahertz Spectroscopy in Biomolecule Detection. Front. Lab. Med..

[16] Weisenstein C., Wigger A.K., Richter M., Sczech R., Bosserhoff A.K., Bolívar P.H. (2021). THz Detection of Biomolecules in Aqueous Environments—Status and Perspectives for Analysis Under Physiological Conditions and Clinical Use. J. Infrared Millim. Terahertz Waves.

[17] Xu J., Plaxco K.W., Allen S.J. (2006). Absorption Spectra of Liquid Water and Aqueous Buffers between 0.3 and 3.72 THz. J. Chem. Phys..

[18] Afsah-Hejri L., Akbari E., Toudeshki A., Homayouni T., Alizadeh A., Ehsani R. (2020). Terahertz Spectroscopy and Imaging: A Review on Agricultural Applications. Comput. Electron. Agric..

[19] Born N., Behringer D., Liepelt S., Beyer S., Schwerdtfeger M., Ziegenhagen B., Koch M. (2014). Monitoring Plant Drought Stress Response Using Terahertz Time-Domain Spectroscopy. Plant Physiol..

[20] Baldacci L., Pagano M., Masini L., Toncelli A., Carelli G., Storchi P., Tredicucci A. (2017). Non-invasive absolute measurement of leaf water content using terahertz quantum cascade lasers. Plant Methods.

[21] Li B., Zhao X., Zhang Y., Zhang S., Luo B. (2020). Prediction and Monitoring of Leaf Water Content in Soybean Plants Using Terahertz Time-Domain Spectroscopy. Comput. Electron. Agric..

[22] Quemada C., Pérez-Escudero J.M., Gonzalo R., Ederra I., Santesteban L.G., Torres N., Iriarte J.C. (2021). Remote Sensing for Plant Water Content Monitoring: A Review. Remote Sens..

[23] Nie P., Qu F., Lin L., Dong T., He Y., Shao Y., Zhang Y. (2017). Detection of Water Content in Rapeseed Leaves Using Terahertz Spectroscopy. Sensors.

[24] Kamiyoshi K., Kudô A. (1978). Dielectric Relaxation of Water Contained in Plant Tissues. Jpn. J. Appl. Phys..

[25] Borovkova M., Khodzitsky M., Demchenko P., Cherkasova O., Popov A., Meglinski I. (2018). Terahertz Time-Domain Spectroscopy for Non-Invasive Assessment of Water Content in Biological Samples. Biomed. Opt. Express.

[26] Cherkasova O.P., Nazarov M.M., Konnikova M., Shkurinov A.P. (2020). THz Spectroscopy of Bound Water in Glucose: Direct Measurements from Crystalline to Dissolved State. J. Infrared Millim. Terahertz Waves.

[27] Zhang X., Wang P., Wang Y., Hu L., Luo X., Mao H., Shen B. (2022). Cucumber Powdery Mildew Detection Method Based on Hyperspectra-Terahertz. Front. Plant Sci..

[28] Zang Z., Li Z., Wang J., Lu X., Lyu Q., Tang M., Cui H.-L., Yan S. (2023). Terahertz Spectroscopic Monitoring and Analysis of Citrus Leaf Water Status under Low Temperature Stress. Plant Physiol. Biochem..

[29] Theocharis A., Clément C., Barka E.A. (2012). Physiological and Molecular Changes in Plants Grown at Low Temperatures. Planta.

[30] Wei S., Tian B.-Q., Jia H.-F., Zhang H.-Y., He F., Song Z.-P. (2018). Investigation on Water Distribution and State in Tobacco Leaves with Stalks during Curing by LF-NMR and MRI. Dry. Technol..

[31] Zang Z., Li Z., Lu X., Liang J., Wang J., Cui H.-L., Yan S. (2021). Terahertz Spectroscopy for Quantification of Free Water and Bound Water in Leaf. Comput. Electron. Agric..

[32] Yan S., Wei D., Tang M., Shi C., Zhang M., Yang Z., Du C., Cui H.-L. (2016). Determination of Critical Micelle Concentrations of Surfactants by Terahertz Time-Domain Spectroscopy. IEEE Trans. Terahertz Sci. Technol..

[33] Fischer B.M., Hoffmann M., Helm H., Wilk R., Rutz F., Kleine-Ostmann T., Koch M., Jepsen P.U. (2005). Terahertz Time-Domain Spectroscopy and Imaging of Artificial RNA. Opt. Express.

[34] Zhang J., Li W., Cui H.-L., Shi C., Han X., Ma Y., Chen J., Chang T., Wei D., Zhang Y. (2016). Nondestructive Evaluation of Carbon Fiber Reinforced Polymer Composites Using Reflective Terahertz Imaging. Sensors.

[35] Datt B. (1999). Remote Sensing of Water Content in Eucalyptus Leaves. Aust. J. Bot..

[36] Ceccato P., Flasse S., Tarantola S., Jacquemoud S., Grégoire J.-M. (2001). Detecting Vegetation Leaf Water Content Using Reflectance in the Optical Domain. Remote Sens. Environ..

[37] Wang X., Zhao L., Yan B., Shi L., Liu G., He Y. (2016). Morphological and Physiological Responses of Heteropogon Contortus to Drought Stress in a Dry-Hot Valley. Bot. Stud..

[38] Wahid A., Gelani S., Ashraf M., Foolad M. (2007). Heat Tolerance in Plants: An Overview. Environ. Exp. Bot..

[39] Miranda M.T., Da Silva S.F., Moura B.B., Hayashi A.H., Machado E.C., Ribeiro R.V. (2018). Hydraulic redistribution in *Citrus* rootstocks under drought. Theor. Exp. Plant Physiol..

[40] Xu Y., Zeng R., Zhou H., Qiu M., Gan Z., Yang Y., Hu S., Zhou J., Hu C., Zhang J. (2022). Citrus FRIGIDA Cooperates with Its Interaction Partner Dehydrin to Regulate Drought Tolerance. Plant J..

[41] Cui Y., Ouyang S., Zhao Y., Tie L., Shao C., Duan H. (2022). Plant Responses to High Temperature and Drought: A Bibliometrics Analysis. Front. Plant Sci..

[42] Rowland L., Ramírez-Valiente J., Hartley I.P., Mencuccini M. (2023). How Woody Plants Adjust Above- and below-Ground Traits in Response to Sustained Drought. New Phytol..

[43] Şimşek Ö., Isak M.A., Dönmez D., Dalda Şekerci A., İzgü T., Kaçar Y.A. (2024). Advanced Biotechnological Interventions in Mitigating Drought Stress in Plants. Plants.

[44] Sehgal A., Sita K., Kumar J., Kumar S., Singh S., Siddique K.H.M., Nayyar H. (2017). Effects of Drought, Heat and Their Interaction on the Growth, Yield and Photosynthetic Function of Lentil (*Lens culinaris* Medikus) Genotypes Varying in Heat and Drought Sensitivity. Front. Plant Sci..

[45] Schneider J.R., Caverzan A., Chavarria G. (2019). Water deficit stress, ROS involvement, and plant performance. Arch. Agron. Soil Sci..

[46] Wang J., Liu Y., Hu S., Xu J., Nian J., Cao X., Chen M., Cen J., Liu X., Zhang Z. (2022). LEAF TIP RUMPLED 1 Regulates Leaf Morphology and Salt Tolerance in Rice. Int. J. Mol. Sci..

[47] Shenglin W., Jingnan L., Lijun W., Jiabao W., Zengliang Y. (2006). The Stimulation Effects of N+ Ion Beam on Liquorice and Its Influence on Water Stress. Plasma Sci. Technol..

[48] Stefi A.L., Papaioannou V., Nikou T., Halabalaki M., Vassilacopoulou D., Christodoulakis N.S. (2022). Heat and Cold-Stressed Individuals of *Pistacia lentiscus* (Mastic Tree) Do Modify Their Secreting Profile. Plants.

[49] Seleiman M.F., Al-Suhaibani N., Ali N., Akmal M., Alotaibi M., Refay Y., Dindaroglu T., Abdul-Wajid H.H., Battaglia M.L. (2021). Drought Stress Impacts on Plants and Different Approaches to Alleviate Its Adverse Effects. Plants.

[50] Feng X., Ackerly D.D., Dawson T.E., Manzoni S., Skelton R.P., Vico G., Thompson S.E. (2018). The Ecohydrological Context of Drought and Classification of Plant Responses. Ecol. Lett..

[51] Yang Y., Zhou S., Song J., Huang J., Li G., Zhu S. (2018). Feasibility of Terahertz Spectroscopy for Hybrid Purity Verification of Rice Seed. Int. J. Agric. Biol. Eng..

[52] Liu C., Shen W., Yang C., Zeng L., Gao C. (2018). Knowns and Unknowns of Plasma Membrane Protein Degradation in Plants. Plant Sci..

[53] Morais M.C., Ferreira H., Cabral J.A., Gonçalves B. (2023). Differential Tolerance of the Woody Invasive Hakea Sericea to Drought and Terminal Heat Stress. Tree Physiol..

[54] Tsarouhas V. (2000). Application of Two Electrical Methods for the Rapid Assessment of Freezing Resistance in *Salix eriocephala*. Biomass Bioenergy.

[55] Silva E.N., Ferreira-Silva S.L., Fontenele A.D.V., Ribeiro R.V., Viégas R.A., Silveira J.A.G. (2010). Photosynthetic Changes and Protective Mechanisms against Oxidative Damage Subjected to Isolated and Combined Drought and Heat Stresses in *Jatropha curcas* Plants. J. Plant Physiol..

[56] Lemonsu A., Beaulant A., Somot S., Masson V. (2014). Evolution of Heat Wave Occurrence over the Paris Basin (France) in the 21st Century. Clim. Res..

[57] Christidis N., Jones G.S., Stott P.A. (2015). Dramatically Increasing Chance of Extremely Hot Summers since the 2003 European Heatwave. Nat. Clim. Chang..

[58] Li L., Chen G., Yuan M., Guo S., Wang Y., Sun J. (2022). CsbZIP2-miR9748-CsNPF4.4 Module Mediates High Temperature Tolerance of Cucumber Through Jasmonic Acid Pathway. Front. Plant Sci..

[59] Persaud L., Bheemanahalli R., Seepaul R., Reddy K.R., Macoon B. (2022). Low- and High-Temperature Phenotypic Diversity of *Brassica carinata* Genotypes for Early-Season Growth and Development. Front. Plant Sci..

[60] Schulze W.X., Altenbuchinger M., He M., Kränzlein M., Zörb C. (2021). Proteome Profiling of Repeated Drought Stress Reveals Genotype-Specific Responses and Memory Effects in Maize. Plant Physiol. Biochem..

[61] Balla K., Karsai I., Kiss T., Horváth Á., Berki Z., Cseh A., Bónis P., Árendás T., Veisz O. (2021). Single versus Repeated Heat Stress in Wheat: What Are the Consequences in Different Developmental Phases?. PLoS ONE.

[62] Song Z., Yan S., Zang Z., Fu Y., Wei D., Cui H.-L., Lai P. (2018). Temporal and Spatial Variability of Water Status in Plant Leaves by Terahertz Imaging. IEEE Trans. Terahertz Sci. Technol..

