# Supplementary material for: Terahertz Spectroscopy in Assessing Temperature-Shock Effects on Citrus
Source: Sensors (Basel). 2024 Nov 15;24(22):7315. doi: 10.3390/s24227315 (PMC11598718; doi:10.3390/s24227315)
Supplement: Supplementary file 1 [file sensors-24-07315-s001.zip › sensors-3291407-supplementary.pdf]

Supplementary Material

Terahertz Spectroscopy in Assessing Temperature-Shock Effects on Citrus

Junbo Wang[1,2], Ziyi Zang[3], Xiaomei Li[1,2], Dongyun Tang[1,2], Qi Xiao[4], Mingkun Zhang[1,2*]and Shihan Yan[1,2*]

[1]Chongqing Institute of Green and Intelligent Technology, Chinese Academy of Science, Chongqing 400714, China.

[2]Chongqing School, University of Chinese Academy of Sciences, Chongqing 400714, China.

[3]Aerospace Times FeiHong Technology Company Limited, Beijing 100094, China.

[4]Wanzhou Institute for Food and Drug Control, Chongqing Key Laboratory of Development and Utilization of Genuine Medicinal Materials in Three Gorges Reservoir Area, Chongqing 404000, China.

*****Correspondence: zhangmk@cigit.ac.cn; yanshihan@cigit.ac.cn

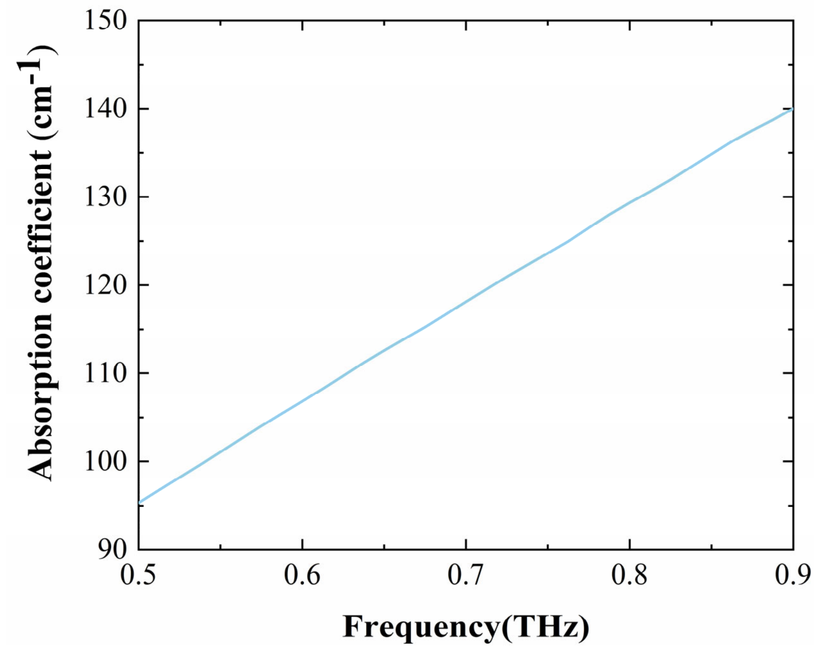

Figure S1. THz absorption coefficient of leaves at room temperature. The absorption coefficient increases monotonically with frequency.

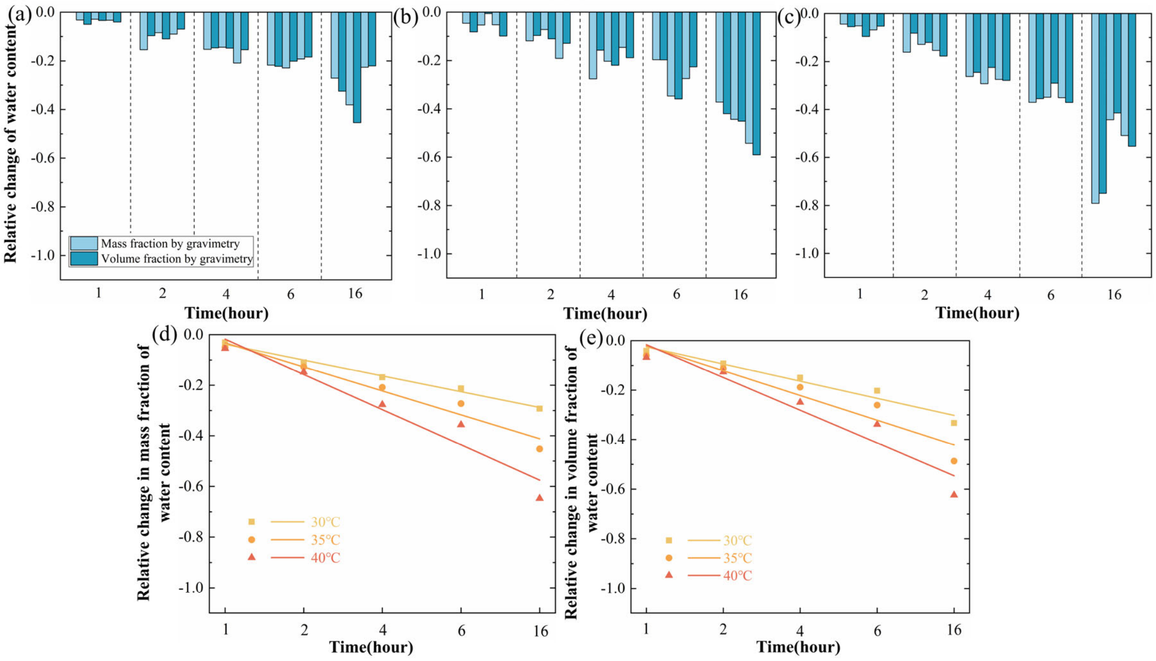

Figure S2. Relative change in water mass fraction and volume fraction measured by the gravimetric method in leaves before and after treatment at 30°C (a), 35°C (b), and 40°C (c) for 1, 2, 4, 6, and 16 h, and linear fitting curves (d, e).

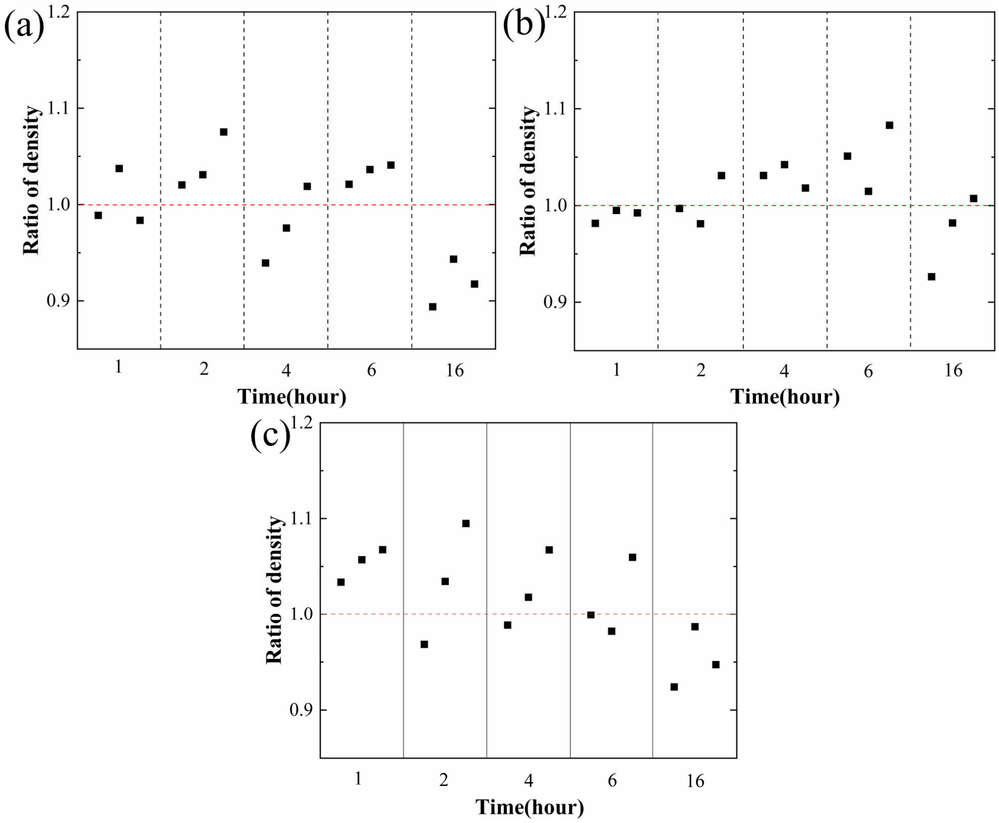

Figure S3. Leaf density ratios before and after treatment at 30°C (a), 35°C (b), and 40°C (c) were derived from the equation and leaf thickness and weight in Table S1.

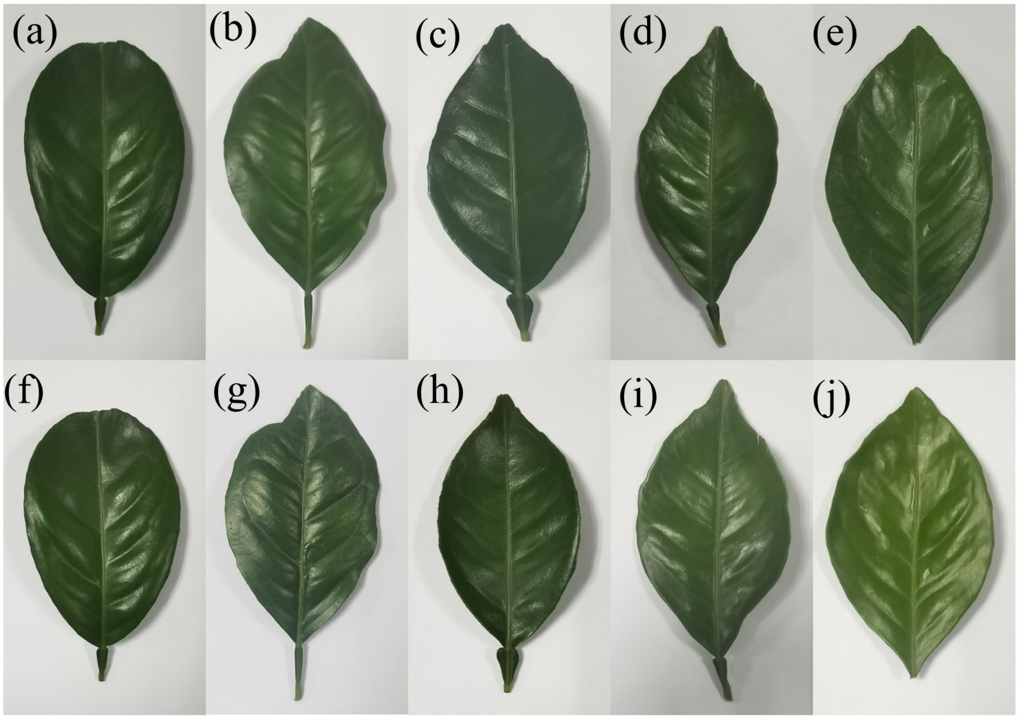

Figure S4. Photos of leaves before (a–e) and after (f–j) treatment at 30°C for 1 hour (a and f), 2 hours (b and g), 4 hours (c and h), 6 hours (d and i), and 16 hours (e and j). There was inconspicuous curling of the blades for the first 6 hours, and 16 hours of high-temperature treatment showed severe damage to the blades.

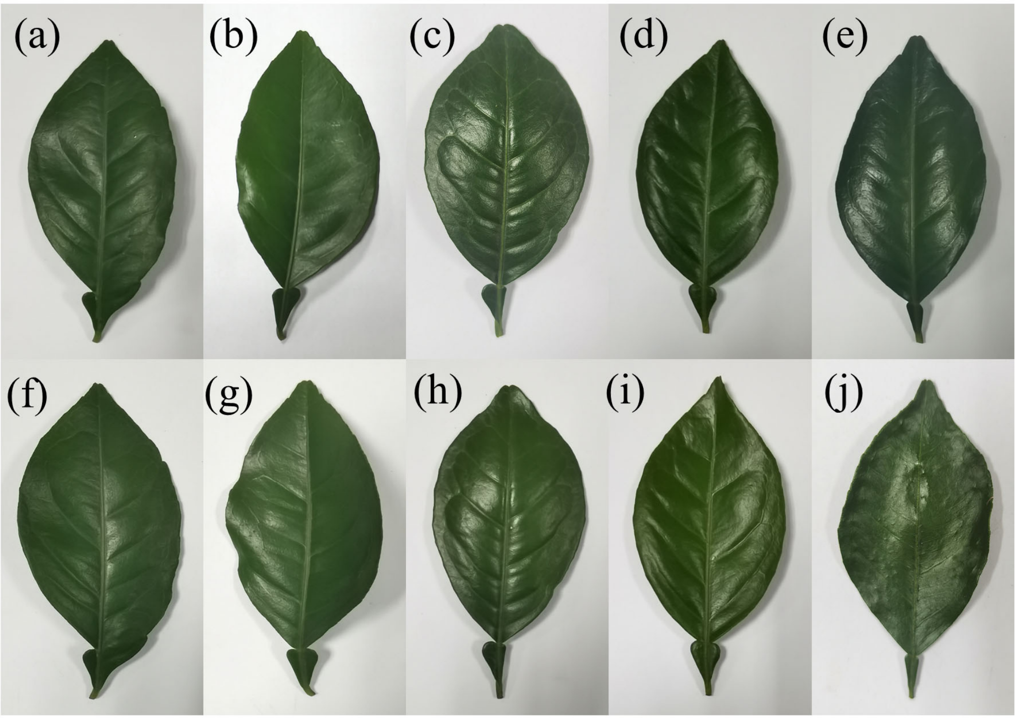

Figure S5. Photos of leaves before (a–e) and after (f–j) treatment at 35°C for 1 hour (a and f), 2 hours (b and g), 4 hours (c and h), 6 hours (d and i), and 16 hours (e and j). There was inconspicuous curling of the blades for the first 6 hours, and 16 hours of high-temperature treatment showed severe damage to the blades.

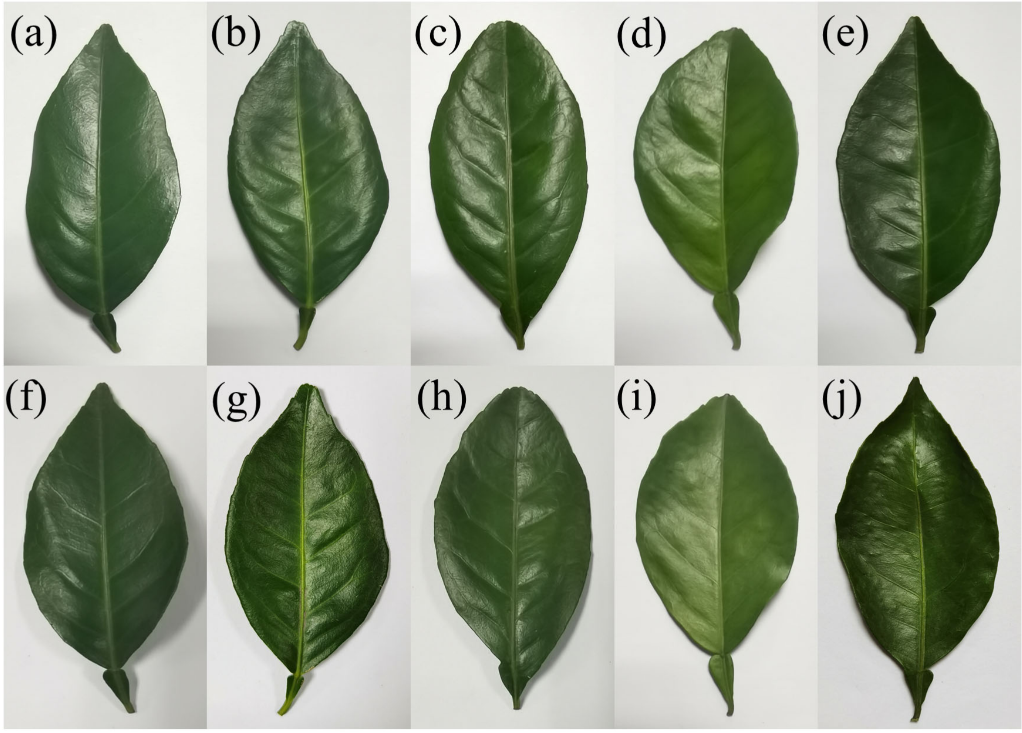

Figure S6. Photos of leaves before (a–e) and after (f–j) treatment at 40°C for 1 hour (a and f), 2 hours (b and g), 4 hours (c and h), 6 hours (d and i), and 16 hours (e and j). There was inconspicuous curling of the blades for the first 6 hours, and 16 hours of high-temperature treatment showed severe damage to the blades.

Table S1. Thickness and weight of leaves before and after high-temperature treatment.

Leaf number	Temperature (°C)	Time (hours)	Thickness			Weight		
			Before treatment (mm)	After treatment (mm)	Relative change amount	Before treatment (g)	After treatment (g)	Relative change
**1-1**	**30**	**1**	0.32	0.30	-0.06	0.77	0.72	-0.06
**1-2**	**30**	**1**	0.34	0.32	-0.06	0.82	0.77	-0.06
**1-3**	**30**	**1**	0.36	0.34	-0.6	0.80	0.76	-0.05
**2-1**	**30**	**2**	0.40	0.32	-0.2	1.50	1.21	-0.19
**2-2**	**30**	**2**	0.38	0.29	-0.24	0.76	0.58	-0.24
**2-3**	**30**	**2**	0.38	0.28	-0.26	1.24	0.96	-0.22
**3-1**	**30**	**4**	0.40	0.29	-0.27	0.97	0.72	-0.26
**3-2**	**30**	**4**	0.39	0.29	-0.26	0.79	0.62	-0.22
**3-3**	**30**	**4**	0.37	0.26	-0.30	0.64	0.46	-0.28
**4-1**	**30**	**6**	0.36	0.28	-0.22	0.72	0.59	-0.18
**4-2**	**30**	**6**	0.34	0.26	-0.24	0.68	0.55	-0.19
**4-3**	**30**	**6**	0.41	0.27	-0.34	0.70	0.50	-0.28
**5-1**	**30**	**16**	0.35	0.24	-0.32	1.10	0.70	-0.36
**5-2**	**30**	**16**	0.35	0.24	-0.32	0.80	0.47	-0.41
**5-3**	**30**	**16**	0.35	0.23	-0.30	0.95	0.66	-0.31
**6-1**	**35**	**1**	0.44	0.36	-0.18	1.00	0.92	-0.08
**6-2**	**35**	**1**	0.37	0.31	-0.16	1.10	0.96	-0.13
**6-3**	**35**	**1**	0.34	0.32	-0.05	0.60	0.53	-0.11
**7-1**	**35**	**2**	0.37	0.30	-0.19	1.17	0.93	-0.20
**7-2**	**35**	**2**	0.37	0.31	-0.16	0.74	0.65	-0.12
**7-3**	**35**	**2**	0.39	0.30	-0.23	0.86	0.72	-0.16
**8-1**	**35**	**4**	0.41	0.29	-0.29	1.02	0.69	-0.32
**8-2**	**35**	**4**	0.39	0.25	-0.36	0.96	0.62	-0.35
**8-3**	**35**	**4**	0.35	0.25	-0.28	0.79	0.62	-0.22
**9-1**	**35**	**6**	0.40	0.29	-0.27	0.69	0.50	-0.28
**9-2**	**35**	**6**	0.36	0.23	-0.36	0.71	0.45	-0.37
**9-3**	**35**	**6**	0.39	0.26	-0.33	0.79	0.56	-0.29
**10-1**	**35**	**16**	0.35	0.19	-0.46	0.80	0.40	-0.50
**10-2**	**35**	**16**	0.36	0.20	-0.44	0.78	0.42	-0.46
**10-3**	**35**	**16**	0.37	0.19	-0.48	1.00	0.46	-0.54

**11-1**	**40**	**1**	0.30	0.28	-0.06	0.83	0.76	-0.08
**11-2**	**40**	**1**	0.33	0.32	-0.03	0.95	0.88	-0.07
**11-3**	**40**	**1**	0.35	0.33	-0.06	0.72	0.66	-0.08
**12-1**	**40**	**2**	0.34	0.30	-0.12	0.67	0.60	-0.11
**12-2**	**40**	**2**	0.35	0.28	-0.20	0.95	0.78	-0.18
**12-3**	**40**	**2**	0.37	0.31	-0.16	0.79	0.71	-0.10
**13-1**	**40**	**4**	0.38	0.29	-0.24	0.75	0.58	-0.23
**13-2**	**40**	**4**	0.39	0.32	-0.18	0.92	0.74	-0.20
**13-3**	**40**	**4**	0.41	0.33	-0.20	0.80	0.62	-0.23
**14-1**	**40**	**6**	0.31	0.20	-0.35	0.78	0.51	-0.35
**14-2**	**40**	**6**	0.43	0.26	-0.40	1.16	0.74	-0.36
**14-3**	**40**	**6**	0.40	0.29	-0.27	0.98	0.73	-0.25
**15-1**	**40**	**16**	0.38	0.21	-0.44	1.32	0.53	-0.59
**15-2**	**40**	**16**	0.38	0.20	-0.47	1.21	0.61	-0.50
**15-3**	**40**	**16**	0.34	0.20	-0.41	1.07	0.50	-0.53
